# Analysis of Neurogenesis during Experimental Autoimmune Encephalomyelitis Reveals Pitfalls of Bioluminescence Imaging

**DOI:** 10.1371/journal.pone.0118550

**Published:** 2015-03-17

**Authors:** Ilya Ayzenberg, Sibylle Schlevogt, Judith Metzdorf, Sarah Stahlke, Xiomara Pedreitturia, Anika Hunfeld, Sebastien Couillard-Despres, Ingo Kleiter

**Affiliations:** 1 Department of Neurology, St. Josef-Hospital, Ruhr-University, Bochum, Germany; 2 Department of Animal Physiology, Ruhr-University, Bochum, Germany; 3 Institute of Experimental Neuroregeneration, Paracelsus Medical University, Salzburg, Austria; 4 Spinal Cord Injury and Tissue Regeneration Center Salzburg (SCI-TReCS), Paracelsus Medical University, Salzburg, Austria; University of Utah, UNITED STATES

## Abstract

Bioluminescence imaging is a sensitive approach for longitudinal neuroimaging. Transgenic mice expressing luciferase under the promoter of doublecortin (DCX-luc), a specific marker of neuronal progenitor cells (NPC), allow monitoring of neurogenesis in living mice. Since the extent and time course of neurogenesis during autoimmune brain inflammation are controversial, we investigated neurogenesis in MOG-peptide induced experimental allergic encephalomyelitis (EAE) using DCX-luc reporter mice. We observed a marked, 2- to 4-fold increase of the bioluminescence signal intensity 10 days after EAE induction and a gradual decline 1–2 weeks thereafter. In contrast, immunostaining for DCX revealed no differences between EAE and control mice 2 and 4 weeks after immunization in zones of adult murine neurogenesis such as the dentate gyrus. *Ex vivo* bioluminescence imaging showed similar luciferase expression in brain homogenates of EAE and control animals. Apart from complete immunization including MOG-peptide also incomplete immunization with complete Freund´s adjuvant and pertussis toxin resulted in a rapid increase of the *in vivo* bioluminescence signal. Blood-brain barrier (BBB) leakage was demonstrated 10 days after both complete and incomplete immunization and might explain the increased bioluminescence signal *in vivo*. We conclude, that acute autoimmune inflammation in EAE does not alter neurogenesis, at least at the stage of DCX-expressing NPC. Effects of immunization on the BBB integrity must be considered when luciferase is used as a reporter within the CNS during the active stage of EAE. Models with stable CNS-restricted luciferase expression could serve as technically convenient way to evaluate BBB integrity in a longitudinal manner.

## Introduction

Bioluminescence imaging (BLI) is a sensitive, easy to perform, and cost-effective approach for neuroimaging [1]. An important advantage of BLI is the possibility of longitudinal *in vivo* measurements. Various transgenic mice with CNS-specific luciferase expression have been used in models of neurodegenerative and autoimmune diseases, particularly experimental allergic encephalomyelitis (EAE) [1–3]. Luo et al. reported that BLI in GFAP-luciferase mice reflects astrocyte activation and precedes the peak clinical score in EAE [3]. Furthermore, BLI was successfully performed to visualize luciferase-transduced neuronal or glial precursor cells after transplantation into the CNS of mice with EAE [4,5]. In contrast to detection based on iron-oxide nanoparticles, BLI monitors only viable cells *in vivo* [6].

Adult neurogenesis, the generation of newborn neurons, can be investigated with BLI as well. A specific marker transiently expressed in neuronal progenitor cells (NPC) is doublecortin (DCX) [7]. Transgenic mice expressing the firefly luciferase (luc) under the DCX promoter (DCX-luc) allow visualization and longitudinal evaluation of neurogenesis in living animals [8].

It is now established that inflammation in the CNS modulates proliferation and differentiation of NPCs. While stimulation of the innate immune systems seems to inhibit neurogenesis [9], others have reported that myelin-specific T cells foster oligodendrogenesis [10] and neurogenesis [11]. Differences in experimental models, immunization protocols and investigated time points are likely at the origin of discrepancies in recent studies investigating adult neurogenesis during EAE [12–16].

The primary aim of our study was to investigate changes of adult neurogenesis in EAE using longitudinal *in vivo* bioluminescence imaging in transgenic DCX-luc mice and to validate findings by immunohistochemistry.

## Methods

### Animals and EAE induction

Eight to 10-week-old B6(Cg)-Tyrc-2J/J DCX-luc reporter mice [8] were immunized by subcutaneous injection with 200 μg myelin oligodendrocyte glycoprotein (MOG)_35–55_ peptide (Charité, Berlin, Germany) in complete Freund's adjuvant (CFA) containing 200 μg *mycobacterium tuberculosis* (Difco). At the day of immunization and 2 days later mice were injected intraperitoneally with 400 ng of pertussis toxin (PTX) (Calbiochem). Control mice received an incomplete immunization without MOG_35–55_, or phosphate buffered saline (PBS). Mice were weighed and scored for clinical signs according to a 5-scale score as described previously [17]. Animal experiments were approved by the local animal use and care committee (The North Rhine-Westphalia State Environment Agency) and were conducted according to all applicable laws.

### Bioluminescence imaging

For BLI the IVIS Lumina II Imaging System (PerkinElmer, USA) and established protocols were used [18]. Briefly, mice were injected intraperitoneally with D-Luciferin (150 mg/kg; Synchem, Germany) and anesthetized with 2% isoflurane. Serial images were taken from 5 to 20 minutes post injection (acquisition time: 59 sec, f-stop: 1, binning: 2, field of view: D). The photon flux (photons/s/cm^2^/steridian) was calculated for the head region that was kept constant in area and positioning, using the LIVINGIMAGE software (PerkinElmer, USA). The maximum photon emission was determined from the acquisition of the signal-time curve, recorded with 59 sec temporal resolution, and corrected for background. Prior to each experiment, two separate baseline measurements were done, the average calculated, and mice stratified for treatment groups according to their average maximum photon flux.

For the *ex vivo* measurement of luciferase activity, fresh hemibrains were weighed and homogenized in 1 mL of Cell Lysis Reagent (Promega, USA). Afterwards tissue homogenates were diluted (1:50) and lysed for 20 minutes in the same lysis reagent. Luciferase activity from tissue homogenates was measured in excess of D-Luciferin and ATP using the Luciferase assay reagent II kit according to the manufacturer´s protocol (Promega, USA). Images were recorded with the IVIS Lumina II Imaging System (acquisition time: 10 sec; f-stop: 1; binning 1; field of view: A) and normalized to weight.

### Immunohistochemistry

At day 14 post immunization, mice were deeply anaesthetized with pentobarbital and transcardially perfused with 4% paraformaldehyde. Brains were removed, immersed in paraformaldehyde for 24 h at 4°C and transferred into 30% sucrose. Serial 20 μM frozen sagittal sections were obtained for histological analysis. Each tenth section was stained with a primary antibody against DCX (1:200, Santa Cruz Biotechnology), a fluorescent secondary antibody (ALEXA Fluor488, Life Technologies), and counterstained with DAPI. The total number of DCX positive cells per dorsal dentate gyrus (DG) was calculated by a blinded rater using fluorescence microscopy (Olympus BX51, Germany). In the subventricular zone (SVZ) and rostral migratory stream (RMS) the total area of DCX-positive cells (pixel^2^) was quantitatively analyzed with ImageJ 1.47v.

### Evans blue assay

Experiments were done in deep pentobarbital anaesthesia. Evans blue dye (EBD), prepared as a 4% solution in 0.9% saline, was injected as a single bolus dose of 4 mL/kg of body weight into the jugular vein. The dye was allowed to circulate for 20 min. Immediately afterwards the mice were transcardially perfused with excess of phosphate-buffered saline (PBS) to remove remaining dye. Perfused brains were divided into both hemispheres. For extraction, the right hemisphere was homogenized in 800 μl 50% trichloroacetic acid by sonication and incubated over night at 4°C. The extracts were centrifuged at 13.200 rpm for 30 min to remove tissue debris and precipitates. Evans blue stain was measured in the supernatant with a spectrophotometer (Eppendorf, Germany) at 600 nm and quantified according to a standard curve. The left hemispheres were collected in 4% PFA for fixation. After 24 h incubation, the samples were transferred into 30% sucrose and serial 30 μM sagittal cryosections were sliced for histological analysis with a fluorescence microscope (Olympus BX51, Germany).

### Statistical analysis

All data are shown as mean ± SEM. Comparisons between groups were done with Mann-Whitney *U* test or ANOVA (Kruskal-Wallis), p<0.05 was considered to be significant. GraphPad Prism 6.0 (GraphPad Software, USA) was used for statistical analysis.

## Results

### Visualization of neurogenesis during EAE using *in vivo* bioluminescence imaging and immunostaining for DCX

**Fig 1:**
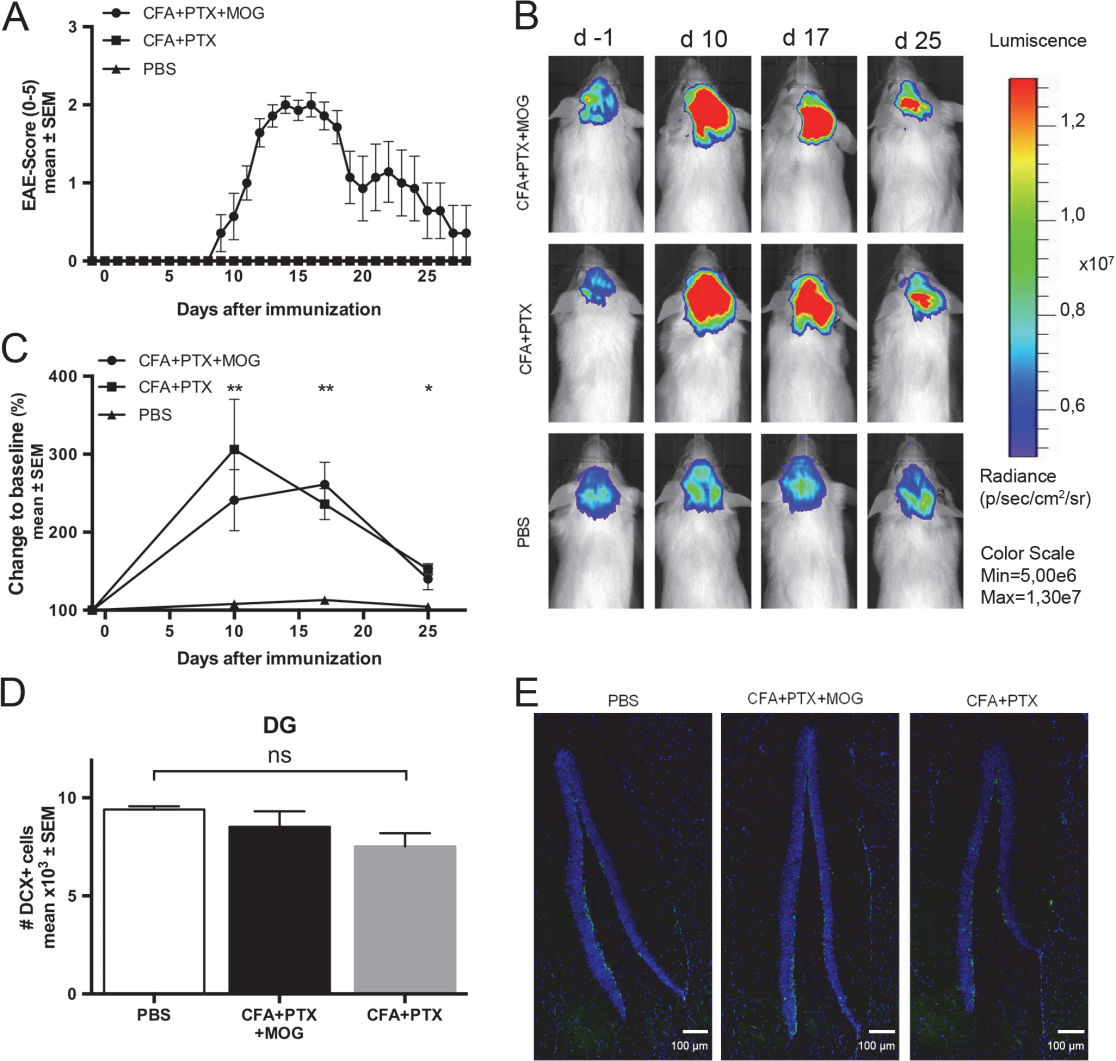
*In vivo* bioluminescence imaging of neurogenesis in mice with EAE and after incomplete immunization. (A) Experimental autoimmune encephalomyelitis (EAE) was induced in doublecortin (DCX)-luc reporter mice by immunization with 200 μg MOG_35–55_ peptide emulsified in CFA+PTX (n = 7). Control mice received incomplete immunization (CFA PTX; n = 5) or PBS (n = 6). The clinical course of EAE was followed for 28 days. (B and C) Bioluminescence of the brain was recorded from minute 5 to 20 after i.p. injection of 150 mg/kg D-Luciferin. The maximum photon flux integrated over 59 seconds is shown. (B) illustrates representative examples, (C) temporal changes of the average bioluminescence signal at 4 time points. Mean + SEM is shown. *p<0.05, **p<0.01 (ANOVA). (D and E) Neurogenesis in the dentate gyrus (DG) 28 days after immunization. DCX positive cells in the DG were counted and presented as the total number of positive cells per hemisphere (mean ± SEM; statistical analysis by ANOVA).

To analyze neurogenesis after induction of EAE in a longitudinal manner, we used the DCX-luc system [8]. We observed a rapid and significant increase of the bioluminescence signal intensity approximately two weeks after antigen-specific immunization (CFA+PTX+MOG), which peaked with the clinical score and declined thereafter (group differences, day 10: p = 0.007, day 17: p = 0.009, day 25: p = 0.031) (Fig. 1A-C). In order to assess the specificity of this effect for EAE, a control group was immunized with complete Freund´s adjuvant and pertussis toxin only (CFA+PTX). Although these mice as anticipated remained clinically unaffected, the recorded bioluminescence unexpectedly increased to the same extent as seen in completely immunized mice (maximal change to baseline: PBS 113%, CFA+PTX 306%, CFA+PTX+MOG 260%). Four weeks after immunization, a small increase of the bioluminescence signal in both immunized groups compared to PBS-injected mice was still observed. However, this difference could not be detected by immunostaining for DCX four weeks after immunization in the dentate gyrus (DG), a neurogenic niche of the CNS (cells/hemisphere, mean ± SD: PBS 9403 ± 166, CFA+PTX 7528 ± 667, CFA+PTX+MOG 8520 ± 793, p = 0.104) (Fig. 1D, E).

### Comparison of *in vivo* and *ex vivo* DCX-driven luciferase activity

**Fig 2:**
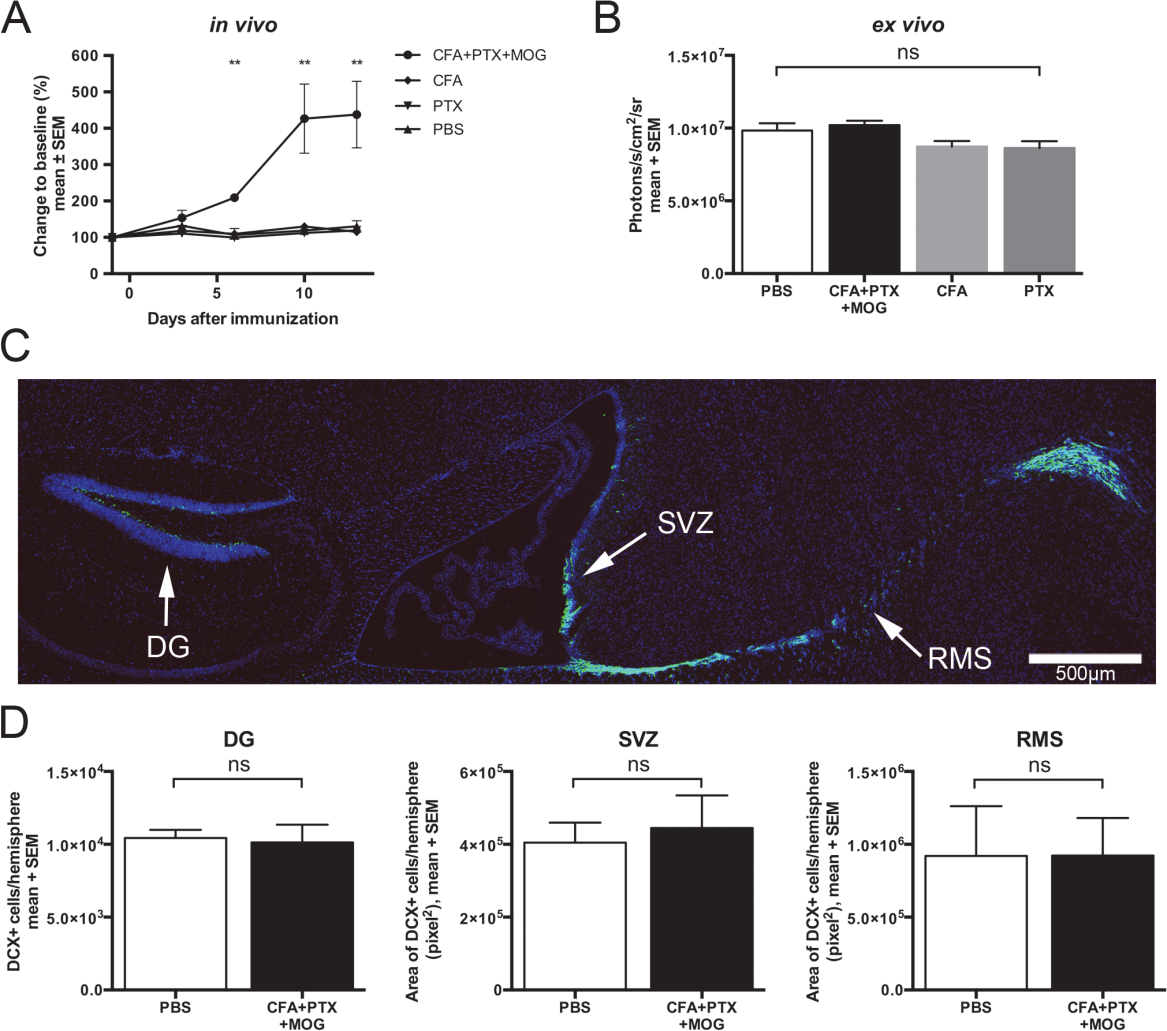
DCX-expression and *ex vivo* bioluminescence remain constant despite increased *in vivo* bioluminescence after EAE induction. (A) *In vivo* bioluminescence imaging of the brain recorded from minute 5 to 20 after i.p. injection of 150 mg/kg D-Luciferin in DCX-luc mice with antigen-specific (200 μg MOG_35–55_+CFA+PTX, n = 6) or incomplete (CFA only, n = 4; PTX only, n = 4) immunization and in the PBS control group (n = 5). The maximum photon flux integrated over 59 seconds is shown. Only mice with antigen-specific immunization developed clinical signs of EAE (not shown). Mice were sacrificed after 14 days and brains were used for *ex vivo* detection of luciferase activity. (B) Brain hemispheres were homogenized and luciferase activity measured by addition of excess D-Luciferin and ATP. (C and D) DCX-luc mice were immunized as detailed above (PBS, n = 6; CFA+PTX+MOG, n = 6) and perfused after 14 days at the peak of EAE. Sagittal brain sections were immunostained for DCX (shown in green) and analyzed in the dentate gyrus (DG), subventricular zone (SVZ), and rostral migratory stream (RMS). (C) A representative example is shown (PBS-treated). (D) DCX positive cells in the DG were counted and presented as the total number of positive cells per hemisphere. DCX positive cells in the SVZ and the RMS are shown as the total area (pixel^2^) of positive cells per hemisphere. Results are presented as mean ± SEM per group. **p<0.01 (ANOVA in A, B; Mann Whitney *U* test in D).

To validate that the steep rise in bioluminescence 10–14 days after immunization reflected increased expression of the DCX-reporter in the CNS and to determine which compound (CFA or PTX) is critical, we did a second EAE experiment, followed by measurement of luciferase activity in brain tissue homogenates at the peak of disease. Six days after antigen-specific immunization, bioluminescence signal intensity increased 2-fold and reached 4-fold compared to baseline between days 10 and 13 (group differences, day 3: p = 0.304, day 6: p = 0.007, day 10: p = 0.006, day 13: 0.006) (Fig. 2A). In contrast to mice with complete (CFA+PTX+MOG) and incomplete (CFA+PTX) immunization (see Fig. 1), no changes of bioluminescence signal intensity were observed in unimmunized mice and mice injected with CFA or PTX alone. Measurement of *ex vivo* luciferase activity in brain homogenates at day 14 post immunization revealed no significant differences between the experimental groups (photons/s/cm2/sr, mean ± SD: PBS 9.8x10^6^ ± 1.1x10^6^, CFA 8.7x10^6^ ± 0.7x10^6^, PTX 8.6x10^6^ ± 0.9x10^6^, CFA+PTX+MOG 10.2x10^6^ ± 0.7x10^6^, p = 0.074) (Fig. 2B). In line with these results, immunostaining for DCX-positive cells in all major neurogenic niches of the murine CNS failed to show differences between mice with antigen-specific immunization and controls (cells/hemisphere, mean ± SD: *DG*: PBS 10438 ± 560, CFA+PTX+MOG 10128 ± 1219, p = 0.675; pixel^2^/hemisphere, mean ± SD: *SVZ*: PBS 404175 ± 54789, CFA+PTX+MOG 444851 ± 89574, p = 0.771; *RMS*: PBS 919802 ± 342667, CFA+PTX+MOG 921790 ± 257355, p = 0.875) (Fig. 2C, D).

### Disruption of blood-brain-barrier after complete and incomplete immunization for EAE

**Fig 3:**
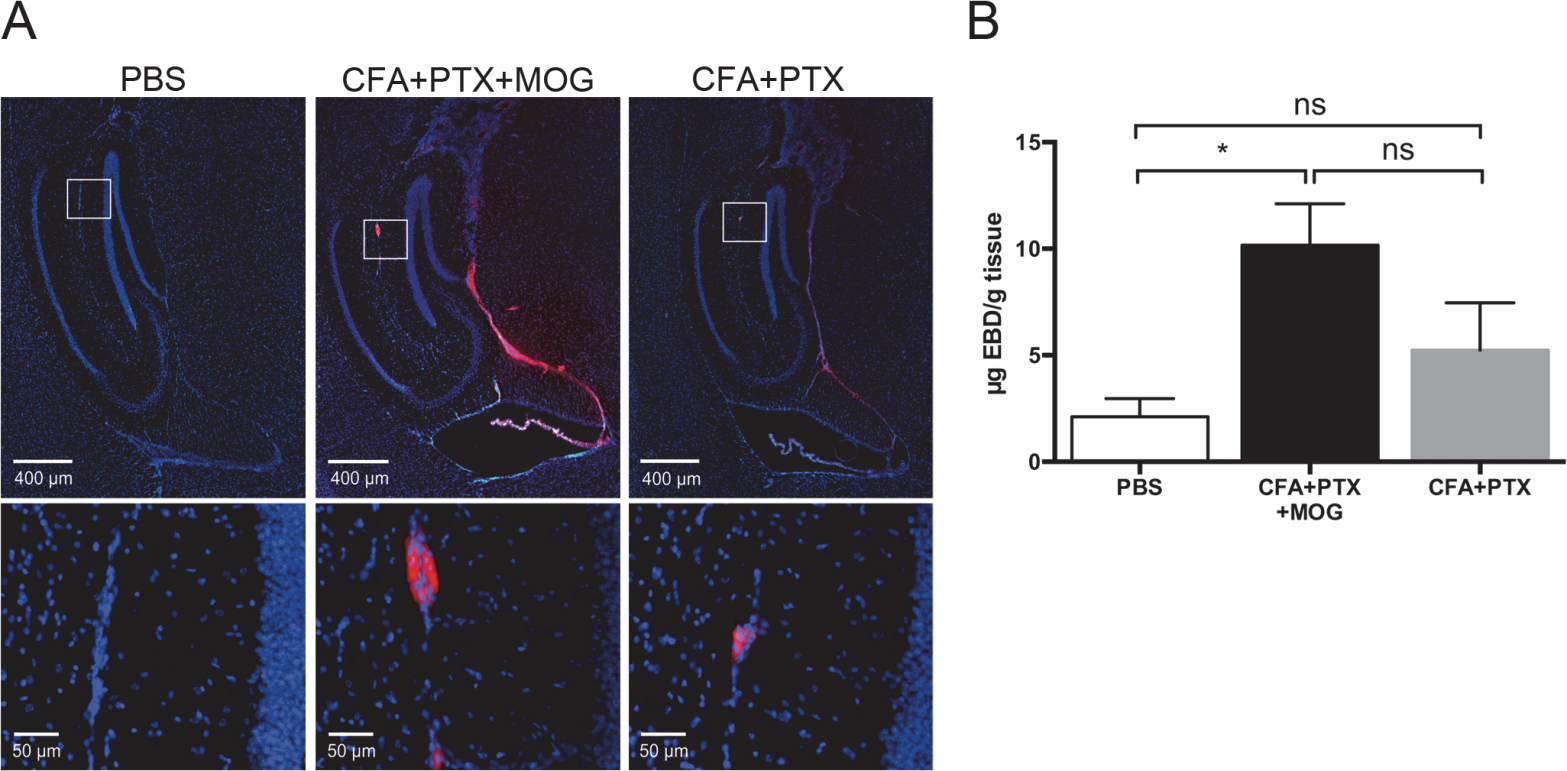
Disruption of the blood-brain-barrier after antigen-specific and incomplete immunization. (A) At day 10 after antigen-specific and incomplete immunization (CFA+PTX+MOG, n = 3; CFA+PTX, n = 4; PBS n = 4) mice were intravenously injected with 4% Evans blue dye (EBD) and sacrificed after 20 minutes of EBD circulation. Fluorescence microscopy revealed an increased blood-brain barrier (BBB) permeability in both immunized groups, most prominent between the DG and the thalamus, in the choroid plexus and around small vessels (insets). (B) Measurement of extracted EBD from brain parenchyma, expressed as μg per g tissue confirms leakage of the BBB after immunization. Results are presented as a mean ± SEM per group. *p<0.05 (ANOVA).

As the bioluminescence signal significantly increased upon EAE induction, but no difference in both immunohistochemical DCX expression and *ex vivo* luciferase activity could be demonstrated, we hypothesized that disruption of the blood-brain barrier (BBB) might contribute to an increased availability of D-Luciferin in the CNS. We used a classical Evans blue assay to analyze BBB integrity at the time point of the BLI-signal peak—ten days after complete and incomplete immunization. In both groups, mice immunized with CFA+PTX+MOG and with CFA+MOG only, a clear disruption of BBB integrity was seen by visual inspection of brains (not shown) und histological analysis (Fig. 3A). Evans blue extravasation was most prominent between the DG and the thalamus, in the choroid plexus and around small vessels. Photometric analysis confirmed that more Evans blue was penetrating in the CNS of mice with complete and incomplete immunization as compared to PBS-injected mice (μg/g brain tissue, mean ± SD: PBS 2.12 ± 1.71, CFA+PTX 5.29 ± 4.45, CFA+PTX+MOG 10.17 ± 3.36, p = 0.041) (Fig. 3B).

## Discussion

Given the neurodegenerative nature of multiple sclerosis from early disease stages on, we intended to investigate whether autoimmune CNS inflammation alters the extent of adult neurogenesis in neurogenic niches of the brain. To be able to evaluate effects of inflammation on NPC in a longitudinal manner, we used *in vivo* BLI in DCX-luc reporter mice [8], followed by immunostaining for DCX. Although we found an early increase of bioluminescence signal intensity in the brains of EAE mice paralleling clinical disease activity, we could not confirm upregulation of DCX expression by immunostaining. Moreover the increase of bioluminescence signal intensity turned out to be non-specific for EAE, as incomplete immunization resulted in the same strong temporary increase.

Previous studies evaluating the effects of autoimmune CNS inflammation on neurogenesis are heterogeneous in methods and outcomes. Several early reports indicated an increase of NPC proliferation in EAE [12,19]. In contrast, other groups demonstrated reduction of neurogenesis due to persistent brain inflammation [13,14]. Especially LPS-induced overexpression of IL-6 and TNF-alpha seems to compromise proliferation and maturation of newborn neurons [9,20,21]. In a few studies, transient positive effects of neuroinflammation on neurogenesis were reported. Aharoni et al. demonstrated an increase of NPC proliferation shortly after EAE appearance, followed by a decline to levels even below that of non-immunized mice [22]. Huehnchen et al. observed an increase of proliferating NPC in EAE, that however did not result in an increase of mature NeuN positive neurons in the DG [15]. Similar to our results, Giannakopoulou et al. could not demonstrate any significant changes in the number of NPC, however, they reported an increase of newborn radial-glia-like progenitor cells [16]. Taking into account the complex nature of autoimmune CNS inflammation, it is likely that different immune mechanisms and their heterogeneous spatial and temporal appearance have divergent effects on neurogenesis. Another explanation for the conflicting results could be that longitudinal changes of NPC proliferation occur during the course of EAE.

Bioluminescence signal intensity in DCX-luc mice peaked at day 10–14 after immunization, similar to the GFAP-Luc model [3]. Since we could not confirm an increase of DCX expression by immunohistochemistry 14 and 28 days after immunization and opposed to *in vivo* BLI, *ex vivo* luciferase activity was not elevated in the brains of EAE mice, alternative explanations have to be considered.

We propose that disruption of the BBB in inflammatory CNS diseases is responsible for increased permeability for D-Luciferin and results in marked changes of the bioluminescence signal in CNS-restricted luciferase models. In such a case, the intensity of the bioluminescence signal would be determined mostly by availability of the substrate (D-Luciferin) and not the concentration of the enzyme (luciferase). Studies examining the distribution of radioactively labeled D-Luciferin demonstrated its extremely low availability in the brain [23,24]. With intact BBB, the concentration of D-Luciferin detected in the brain is approximately 80-fold lower compared to that of blood and the efficacy of BLI in the brain is approximately 200-fold lower than that in the liver [23–25]. According to Michaelis-Menten kinetics, a decrease in the substrate concentration with a fixed enzyme concentration causes reduction of the reaction rate, and below the Michaelis constant (8 μM for D-Luciferin) this relationship is linear [23,26]. In a recent study with the DCX-luc model, injection of 750 mg/kg D-Luciferin, i.e. 5-times standard concentration used in our and other studies [3], failed to reach signal saturation in the brain and resulted in a 10-fold increase of bioluminescence signal intensity [18].

It is well established that the integrity of the BBB is compromised during the course of EAE, facilitating the transport of soluble and cellular compounds across the BBB [27]. Precise mechanisms of BBB disruption in EAE are still not completely understood. In earlier studies a role for both PTX [28–30] and CFA [31–33] has been shown, and a combination of CFA and PTX has been proven to be optimal for EAE induction. Using a classical Evans blue assay, we could confirm leakage of the BBB in mice with complete and incomplete immunization. Noubade et al. demonstrated an increase of BBB permeability for albumin to a similar extent after antigen-specific and incomplete (CFA+PTX) immunization [34]. The combination of CFA and PTX seems to be critical for BBB disruption, as the immunization with CFA or PTX alone did not result in an increase of the bioluminescence signal intensity in our study as well as in a previous report [3]. In line with these data, Bennett et al. histologically could not demonstrate tight junction disruption after stimulation with CFA or PTX alone [35].

We conclude from our study that the early effects of acute autoimmune CNS inflammation on adult neurogenesis are not perspicuous. Two and four weeks after immunization with CFA+PTX+MOG, we did not detect significant differences in the number of DCX-expressing NPCs within neurogenic regions. The use of simpler inflammatory models, rather than complex autoimmune inflammation as EAE, would at this point be helpful in dissecting immune mechanisms leading to stimulation or decrease of neurogenesis. Secondly, when reporter strains or transplanted cells with CNS-restricted luciferase expression pattern are used for the analysis of inflammatory processes such as EAE, the effects of immunization or treatments on the BBB integrity must be taken into account. Validation measurements of the CNS luciferase activity *ex vivo* is compulsory to detect a false signal increase caused by BBB disruption. On the other side, the use of BLI in models with constitutive luciferase expression in the CNS might constitute as convenient way to evaluate BBB integrity in a longitudinal manner.
